# Thymic homing of activated CD4^+^ T cells induces degeneration of the thymic epithelium through excessive RANK signaling

**DOI:** 10.1038/s41598-017-02653-9

**Published:** 2017-05-25

**Authors:** Chen Yin, Xiao-Yan Pei, Hui Shen, Ya-Nan Gao, Xiu-Yuan Sun, Wei Wang, Qing Ge, Yu Zhang

**Affiliations:** 1 Department of Immunology, School of Basic Medical Sciences, Key Laboratory of Medical Immunology of Ministry of Public Health, Peking University Health Science Center, Beijing, China; 2 Institute of Biological Sciences, Jinzhou Medical University, Jinzhou, Liaoning China

## Abstract

Activated T cells have been shown to be able to recirculate into the thymus from the periphery. The present study was aimed to elucidate the functional consequences of thymic homing of activated T cells upon developing thymocytes and thymic epithelial cells (TEC). In the presence of activated T cells, especially CD4^+^ T cells, T cell development was found to be inhibited in thymic organ cultures with markedly reduced cellularity. Thymic transplantation demonstrated that the inhibitory effect was most likely due to a defective microenvironment. As the major component of the thymic stroma, the TEC compartment was severely disturbed after prolonged exposure to the activated T cells. In addition to reduced cell proliferation, TEC differentiation was heavily skewed to the mTEC lineage. Furthermore, we demonstrated that RANKL highly expressed by activated CD4^+^ T cells was primarily responsible for the detrimental effects. Presumably, excessive RANK signaling drove overproduction of mTECs and possibly exhaustion of epithelial progenitors, thereby facilitating the deterioration of the epithelial structures. These findings not only reveal a novel activity of activated T cells re-entering the thymus, but also provide a new perspective for understanding the mechanism underlying thymic involution.

## Introduction

The thymus is a primary immune organ responsible for the development of T lymphocytes. Hematopoietic progenitors seeding the thymus undergo proliferation, differentiation, T cell receptor (TCR) gene rearrangement, positive and negative selections, and functional maturation, culminating in the generation of a T cell repertoire capable of responding to a diverse array of foreign antigens but tolerant to self antigens^1, 2^. During this process, T cell precursors migrate through structurally and functionally distinct cortical and medullary regions. The interactions with cortical thymic epithelial cells (cTECs) and medullary thymic epithelial cells (mTECs) provide the signals essential for thymocyte development^3, 4^. CTECs, for example, are the predominant sources of Notch ligands, cytokines and chemokines required for the early differentiation of T cell precursors. In addition, cTECs play an important role in positive selection by generating a distinct set of self-peptides through their unique antigen processing machinery^5–7^. MTECs, on the other hand, mediate negative selection via ectopic expression of tissue-restricted antigens driven by Aire or Fezf2^8, 9^.

As a rather dynamic population, thymic epithelial cells (TECs) are rapidly replaced every few weeks^10^. Such a high rate of turnover requires continuous input from a progenitor pool. A recent study by Ucar *et al*. has reported the identification of a CD45^−^ EpCam^−^ Sca-1^+^ CD24^−^ FoxN1^−^ TEC subset, which has self-renewing activities and the capacity to differentiate into both the cTEC and mTEC lineages^11^. Wong *et al*., however, have shown that the bipotent progenitors are highly enriched in a CD45^−^ FoxN1^+^ EpCam^+^ UEA-1^−^ MHCII^low^ α6-integrin^high^ Sca-1^high^ TEC population^12^. Regarding the events downstream of the bipotent progenitors, while limited information is available for the cTEC lineage, the developmental pathway of mTEC has been partially resolved. The earliest mTEC-restricted progenitors are characterised by the expression of SSEA-1, claudin 3 and claudin 4^13^. Further downstream are three successive stages, defined as immature MHCII^low^ CD80^low^ Aire^−^ mTECs, mature MHCII^high^ CD80^high^ Aire^+^ mTECs, and terminally differentiated MHCII^low^ CD80^low^ Aire^low^ Involucrin^+^ mTECs^14, 15^. Interestingly, mTECs not only provide key signals for T cell differentiation but also rely on the interactions with thymocytes for their own development^16–18^. These interactions are primarily mediated by the tumor necrosis factor receptor family (TNFRF) members, including receptor activator of NF-κB (RANK), CD40, and lymphotoxin-β receptor (LTβR)^17, 19–23^. Upon binding to the corresponding ligands expressed on developing thymocytes, these receptors signal the activation of NF-κB transcription factors, which are known to play a crucial role in the regulation of mTEC differentiation.

The thymus reaches its maximum size around puberty. After that, it undergoes progressive regression, leading to reduced tissue mass and cellularity, disorganised morphology, and diminished production and exportation of naive T cells^24–26^. This process – known as thymic involution – is one of the most dramatic and ubiquitous changes in the ageing immune system and represents a major mechanism for the age-related decline in immune function^26^. The physiological significance of this seemingly undesired process remains elusive. Presumably, it may help to reduce the risk of leukemia, to enhance peripheral selection, or to convert energy to other important biological activities, such as reproduction^25, 27^. More recently, we have demonstrated that reduced thymic output actually favours the maintenance of the memory T cell pool^28^. The mechanism behind thymic involution is also a subject of debate. Several hypotheses have been proposed, including ageing hematopoietic progenitors, dysfunctioning thymic microenvironment, and elevating sexual hormone levels^24–27^. Currently, the prevailing view holds that thymic involution primarily results from a deteriorating epithelial compartment^29–31^. However, it remains unsolved why the thymic epithelium starts to deteriorate early in life and how it is initiated.

Although the thymus primarily functions to export mature T cells, it has long been recognised that peripheral T cells are capable of recirculating into the thymus^32–34^. Under normal conditions, re-entry is largely restricted to activated or memory T cells^32, 34^. Once homing to the thymus, they are predominantly localized in the medulla and remain there for prolonged periods^33^. It is estimated that the thymus of an adult mouse can accommodate about 10^5^ recirculating peripheral T cells^35–37^. Despite the relatively constant number, the proportion of such cells increases dramatically with age, accounting for more than 20% of single positive (SP) thymocytes in mice over 1 year of age^37^. Is the presence of peripheral T cells in the thymus merely an epiphenomenon or are they destined to fulfill some relevant physiological functions ? Despite controversy, evidence is emerging that these cells may participate in the shaping of the T cell repertoire by delivering self antigens into the thymus^35, 36, 38–40^.

Using fetal thymic organ cultures (FTOCs), the present study investigated the impact of recirculating T cells on the two major cell populations in the thymus, namely the thymocytes and the TECs. Co-culturing with activated T cells, especially CD4^+^ T cells, had a significant inhibitory effect on T cell development. This effect was most likely due to the structural and functional abnormalities of TECs induced by prolonged exposure to the activated T cells. Furthermore, we demonstrated that the excessive RANK signaling was a major contributor to the deterioration of the epithelial structures. These findings provide a new perspective on the role of recirculating T cells and its implication in age-related thymic involution.

## Results

### Activated CD4^+^ T cells homing to the thymus inhibit T cell development

**Figure 1:**
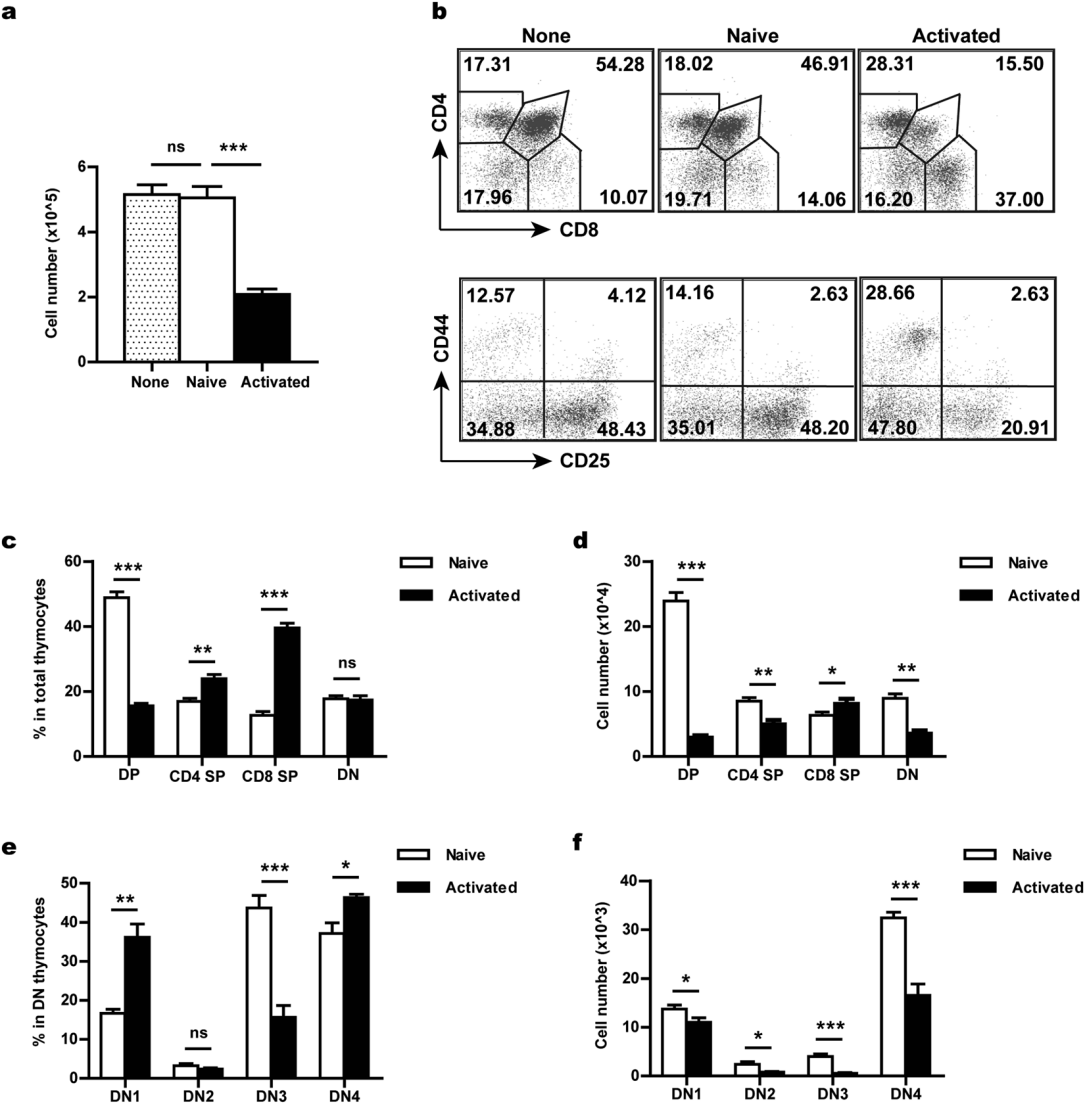
T cell developmental defects in the activated CD4^+^ T cell-treated FTOC. Fetal thymic organ cultures were set up with d16 fetal thymuses (CD45.2^+^) pre-incubated without or with 2 × 10^4^ naive or activated CD4^+^ T cells (CD45.1^+^). The cultures were harvested and analysed at day 12. (**a**) The total number of thymocytes recovered from a single thymic lobe under different culture conditions. (**b**) Flow cytometric analysis of T cell development in FTOCs. Dot plots show representative profiles for CD4/CD8 staining (gated on CD45.2^+^ cells) and for CD25/CD44 staining (gated on CD45.2^+^ CD4^−^ CD8^−^ DN cells). (**c**,**d**) The percentage (**c**) and absolute number (**d**) of DN, DP, CD4 SP and CD8 SP thymocytes in naive or activated T cell-treated cultures. (**e**,**f**) The percentage (**e**) and absolute number (**f**) of different subsets of DN thymocytes in naive or activated T cell-treated cultures. DN1, CD25^−^ CD44^+^; DN2, CD25^+^ CD44^+^; DN3, CD25^+^ CD44^−^; and DN4, CD25^−^ CD44^−^. The experiments were repeated at least six times with 3- 5 thymic lobes for each group in each experiment. Data are presented as Mean ± SEM. *p < 0.05; **p < 0.01; ***p < 0.001; and ns, not significant.

We first sought to explore the potential impact of recirculating T cells on thymopoiesis. Day 16 fetal thymuses were pre-incubated with CD4^+^ T cells stimulated with anti-CD3 and anti-CD28 or an equal number (2 × 10^4^) of naive CD4^+^ T cells in hanging drops for 24 hours. Like the adult thymus, the fetal thymus was permeable to activated but not naive T cells (Supplementary Fig. S1a). Typically, the activated T cells accounted for 10–20% of CD4^+^ SP thymocytes, similar to what has been reported in aged mice^37^. After removal of free cells, FTOCs were established. T cell development was examined at day 6 and 12, two time points each associated with a wave of thymocyte development normally seen in FTOC^41, 42^. The day 6 culture showed a modest reduction of total thymocytes in the activated T cell-treated group (Supplementary Fig. S1d). But the profiles of major thymocyte populations were largely comparable except for a slight decrease of double negative (DN) 3 cells in the activated cell-treated group (Supplementary Fig. S1b,c). On day 12, however, dramatic differences were observed between the two cultures. In addition to a 60% decrease in total thymocytes (Fig. 1a), co-culture with activated CD4^+^ T cells resulted in a remarkable suppression of double positive (DP) cell production, which was accompanied by proportional increases of CD4 and CD8 SP cells (Fig. 1b, upper and c). Among the DN thymocytes, the DN3 subset was found to be markedly diminished, while there was a relative accumulation of DN1 cells (Fig. 1b, lower and e). Of note, no difference was observed between untreated or naive cell-treated cultures, either in the cell number or in the staining profile (Fig. 1a,b). These results indicate that the presence of activated (but not naive) CD4^+^ T cells has a detrimental effect on T cell development.

We next tested whether activated CD8^+^ T cells might have similar suppressive activities. Activated CD8^+^ T cells seeded the fetal thymus as efficiently as activated CD4^+^ T cells (Supplementary Fig. S2a). But they had minimal impact on T cell development. Despite a slight decrease in DP and DN3 cells, similar numbers of total thymocytes were obtained in day 12 cultures treated with naive or activated CD8^+^ T cells (Supplementary Fig. S2b–d). Therefore, the suppressive function is largely restricted to activated CD4^+^ T cells.

### Prolonged exposure to activated CD4^+^ T cells impairs the thymopoiesis-supporting capacity of the thymus *in vivo*

**Figure 2:**
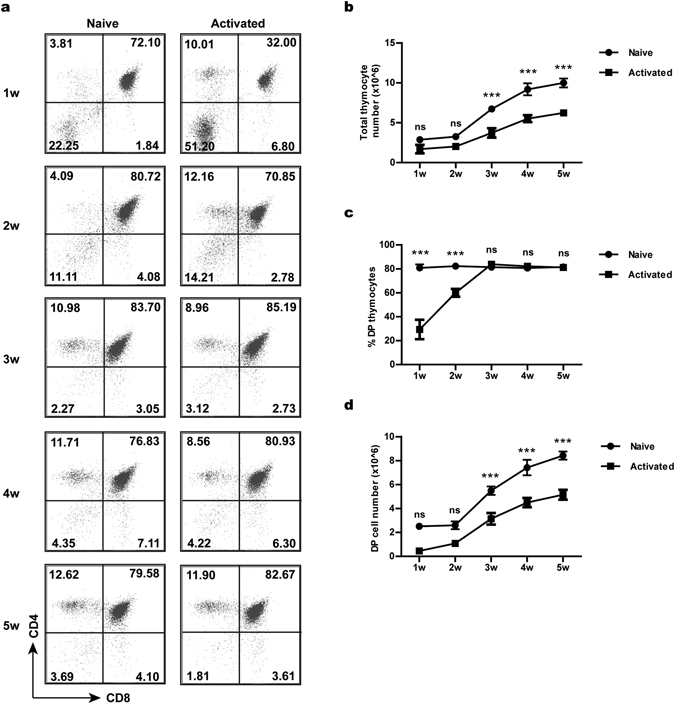
The impaired capacity of activated T cell-treated fetal thymuses to support T cell development *in vivo*. Fetal thymuses (CD45.2^+^) were first mixed with naive or activated T cells in hanging drops for 24 hours and then cultured in FTOC for 12 days before being implanted under the kidney capsules of CD45.1^+^ mice. The development of host T cells in the implants was analysed by flow cytometry. (**a**) Representative CD4/CD8 staining profiles of CD45.1^+^ host-derived thymocytes at different time points after transplantation. The number indicates the percentage of cells in each quadrant. (**b**) Cell counts of host-derived thymocytes. (**c**) The percentage of DP thymocytes. (**d**) The absolute number of DP thymocytes. The experiments were repeated three times with at least 5 mice for each experiment. Data are presented as Mean ± SEM. *p < 0.05; **p < 0.01; ***p < 0.001; and ns, not significant. In addition to the point-to-point comparisons shown in the figure, two-way Anova analysis was performed to determine the statistical significance of the differences between the two groups over the time course in terms of total thymocyte number (p < 0.0001), percentage of DP thymocytes (p < 0.0001) and absolute number of DP thymocytes (p < 0.0001).

The inhibition of T cell development by recirculating T cells could result from either a direct action on developing thymocytes or an indirect effect on thymic microenvironment. The much more severe developmental defect in day 12 than day 6 cultures appears to favour an indirect effect. To clarify this issue, the fetal thymuses from CD45.2^+^ mice were pre-incubated with activated or naive CD4^+^ T cells for 24 hours, cultured for 12 days and then transplanted under the kidney capsule of CD45.1^+^ congenic mice. At different time points, the development of host-derived thymocytes was monitored by gating on CD45.1^+^ cells. A developmental delay was observed in the activated T cell-treated graft (Fig. 2). In contrast to the high percentage (>80%) of DP cells generated in the control graft, less than 30% of host-derived thymocytes proceeded to the DP stage in the activated T cell-treated graft one week after transplantation (Fig. 2a, top). Meanwhile, more than half of the cells were found to be retained in the DN stage (Fig. 2a, top). A largely normal profile was restored at week 3 and afterwards (Fig. 2a). Nevertheless, the number of total thymocytes (Fig. 2b), as well as that of DP cells (Fig. 2d), was constantly lower in the activated T cell-treated thymic graft than the control graft at all time points examined. These data support that the prolonged exposure to activated CD4^+^ T cells may cause permanent changes in the thymic microenvironment, leading to a reduced capacity to support T cell development.

### The composition of the TEC compartment is distorted in activated CD4^+^ T cell-treated FTOCs

**Figure 3:**
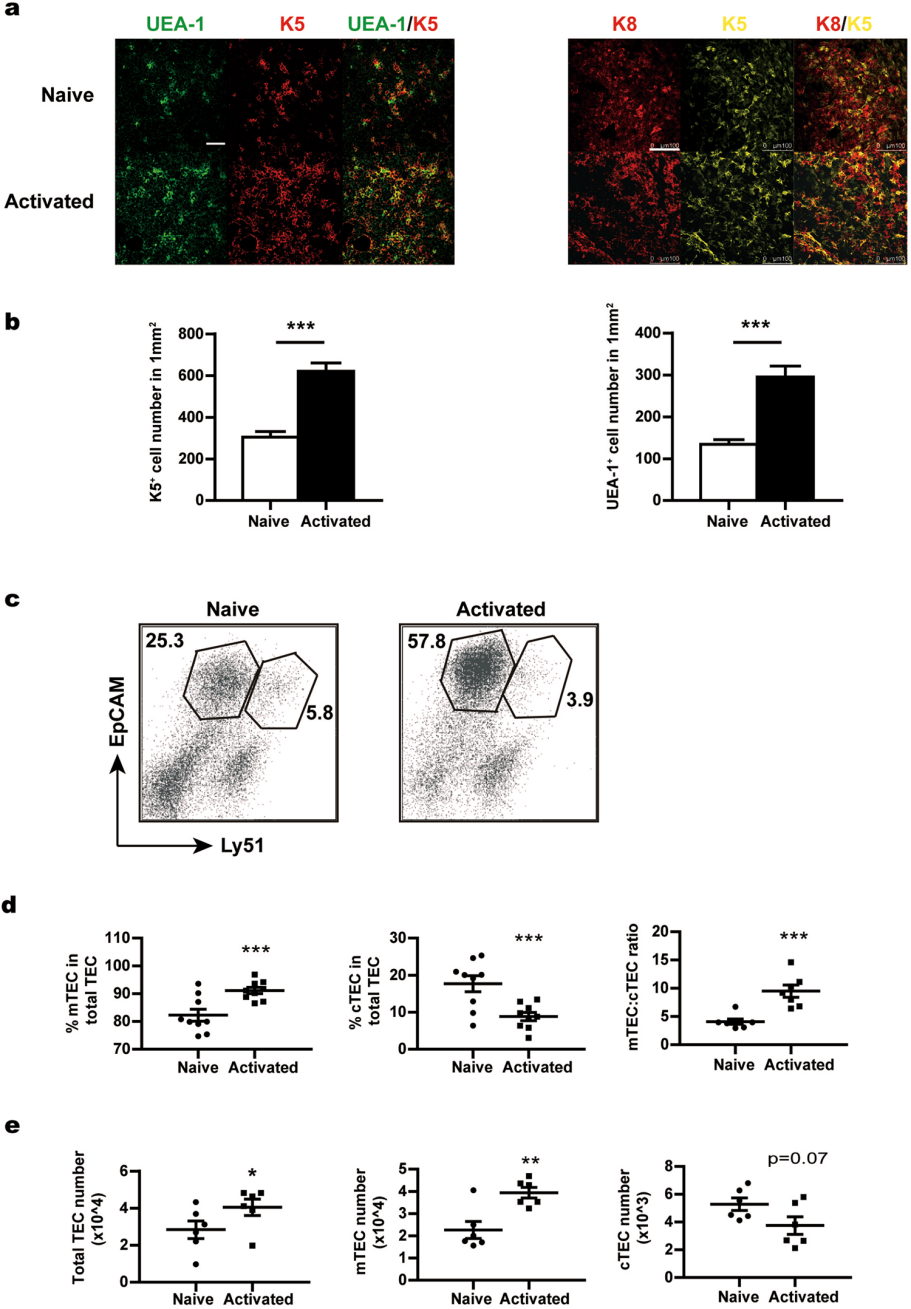
The distorted composition of thymic epithelial cells in the activated T cell-treated fetal thymus. Fetal thymuses were cultured in the presence of naive or activated CD4^+^ T cells and analysed at day 12. (**a**) Thymic lobes were examined by confocal microscopy to detect the expression of keratin 5 (K5) and keratin 8 (K8) and the binding activity for UEA-1. Representative images from three independent experiments are shown. Scale bars: 75 μm for UEA-1 and K5 staining; 100 μm for K8 and K5 staining. (**b**) The numbers of K5^+^ (left) and UEA-1^+^ (right) cells were counted in 10 randomly chosen sections of 370 × 370 μm^2^ and then converted into cell number per mm^2^. Data from three independent experiments are presented as Mean ± SEM. (**c**) Single cell suspension of thymic cells was prepared. MACS-enriched CD45^−^ stromal cells were stained with antibodies against CD45, EpCam and Ly51. Representative dot plots are shown with gates for mTECs (CD45^−^ EpCam^+^ Ly51^−^) and cTECs (CD45^−^ EpCam^+^ Ly51^+^). (**d**) The percentage of mTECs (left) and cTECs (middle) in total TECs and the ratio of mTECs versus cTECs (right). (**e**) The absolute number of total TECs (left), mTECs (middle) and cTECs (right). The experiments were repeated 6–9 times. Each experiment included 20–30 fetal thymic lobes for each group, which were then pooled for analysis. Each dot represents the result from a single experiment. Data are presented as Mean ± SEM. *p < 0.05, **p < 0.01; ***p < 0.001; and ns, not significant.

The TECs provide the specialised and unique microenvironment for T cell development and the degeneration of the thymic epithelium is proposed to be a major mechanism behind thymic involution^25, 26, 29, 43^. Subsequent studies were therefore focused on the impact of activated CD4^+^ T cells on the TEC compartment. Fetal thymuses harvested from day 12 cultures were examined by immunofluorescent staining with antibodies against keratin 5 (K5) and keratin 8 (K8) or with Fluorescein-UEA-1. As shown in Fig. 3a and b, co-cultures with activated T cells resulted in a marked increase of K5^+^ and UEA-1-binding cells. Consistent with these results, flow cytometric analysis revealed that the activated T cell-treated fetal thymus contained a higher percentage of EpCam^+^ Ly51^−^ mTECs but a lower percentage of EpCam^+^ Ly51^+^ cTECs (Fig. 3c and d, left and middle). As such, the ratio of mTECs versus cTECs was increased from 4 in naive T cell-treated thymuses to 10 in activated T cell-treated ones (Fig. 3d, right). In terms of absolute cell number, we saw a significant increase in total TECs and mTECs in activated T cell-treated thymuses (Fig. 3e, left and middle). On the other hand, the number of cTECs was found to be reduced, although not to a level of statistical significance (Fig. 3e, right). Together, these data indicate that the TEC compartment is disturbed upon thymic homing of activated CD4^+^ T cells. Moreover, the activated T cells have a differential effect on the mTEC versus cTEC lineages.

### Activated CD4^+^ T cells induce a generally enhanced production of mTECs

**Figure 4:**
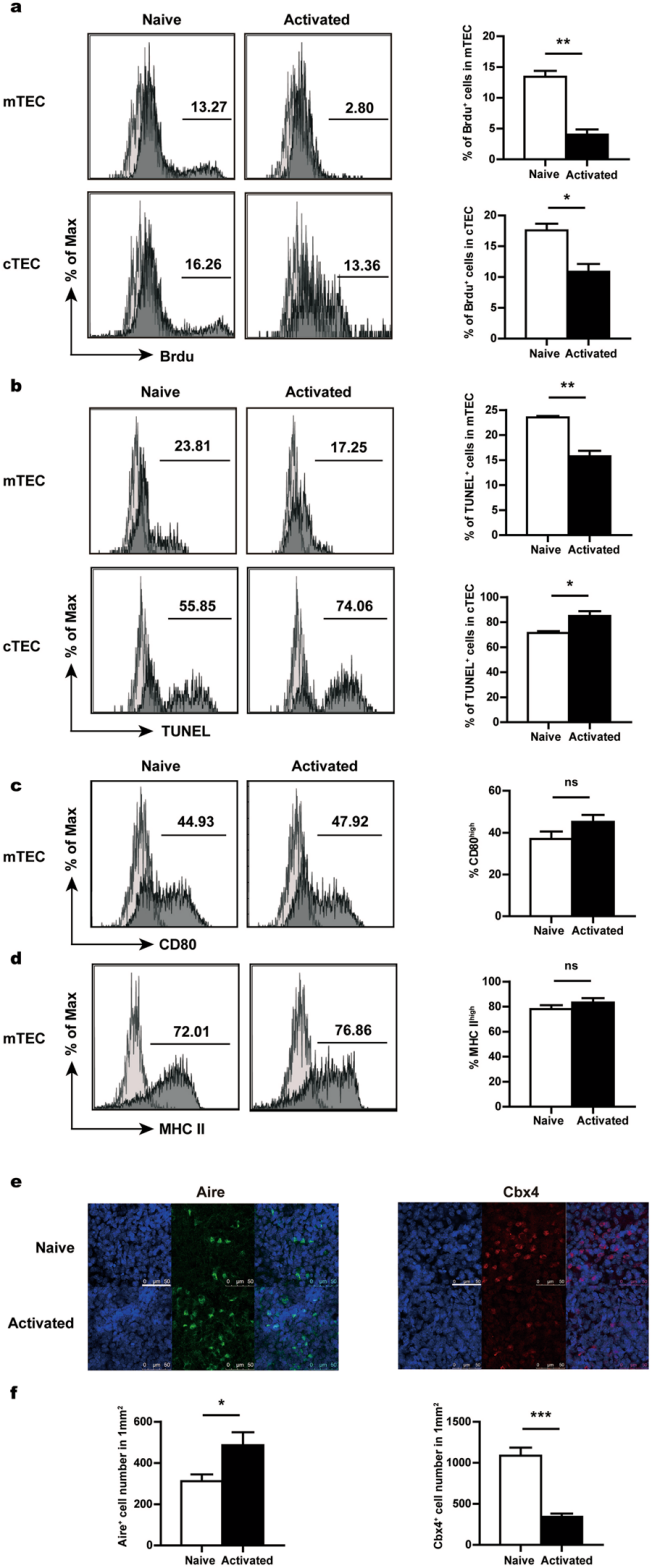
Inhibited TEC proliferation but enhanced mTEC production in the activated T cell-treated fetal thymus. Fetal thymuses were co-cultured with naive or activated CD4^+^ T cells for 12 days. mTECs were gated on CD45^−^ EpCam^+^ Ly51^−^ cells and cTECs were gated on CD45^−^ EpCam^+^ Ly51^+^ cells in flow cytometric analysis. (**a**) Brdu was added to the culture 24 hours before harvesting. Brdu incorporation was detected by flow cytometry. Representative histograms (left) and the percentage of Brdu^+^ cells (right) are shown for mTECs (upper) and cTECs (lower), respectively. (**b**) TUNEL assay was performed to evaluate the apoptosis of mTECs (upper) and cTECs (lower). Representative histograms (left) and the percentage of TUNEL^+^ cells (right) are shown. (**c**) The expression of CD80 in mTECs was measured by flow cytometry. Representative histograms (left) and the percentage of CD80^high^ cells (right) are shown. (**d**) Representative histograms of MHCII staining (left) and the percentage of MHCII^high^ cells (right) in mTECs. Shallow shades represent the staining controls for different markers. (**e**) Immunofluorescent staining was performed to detect the expression of Aire and Cbx4. Co-staining was performed with Hoechst 33342. Scale bars: 50 μm. Similar results were obtained from three independent experiments. Representative images are shown. (**f**) The number of Aire^+^ (left) and Cbx4^+^ (right) cells were counted in 10 randomly chosen sections of 145 × 145 μm^2^ and then converted into cell number per mm^2^. Data from three independent experiments are presented as Mean ± SEM. *p < 0.05; **p < 0.01; p < 0.001; and ns, not significant.

To understand the cellular mechanisms underlying the altered TEC composition, we compared TEC proliferation, apoptosis and differentiation in naive and activated CD4^+^ T cell-treated FTOCs. For the proliferation assay, Brdu was added into the culture medium one day before the harvest of FTOCs on day 12. Epithelial cells were then isolated and assessed for Brdu incorporation using flow cytometry. Intriguingly, in spite of the increased number of total TECs, the proliferation of both mTECs and cTECs was found to be suppressed in activated T cell-treated FTOCs (Fig. 4a). Specifically, the Brdu^+^ cells were reduced by 40–60% in these cultures when compared with the naive T cell-treated cultures (Fig. 4a, right).

The TUNEL assay was next applied to evaluate the apoptosis of TECs. cTECs from FTOC had a high basal level of apoptosis, which was further elevated in the presence of activated T cells (Fig. 4b, lower). On the contrary, mTECs were much less prone to apoptosis and their viability was even slightly enhanced with activated T cells (Fig. 4b, upper). However, it is difficult to envisage that this modestly improved cell survival is fully responsible for the marked increase in the number of mTECs in activated T cell-treated FTOCs.

Lastly, we explored the possibility that the activated CD4^+^ T cells might enhance the differentiation of mTECs. It has been well established that the maturation of mTECs is characterised by a phenotypic conversion from MHCII^low^ CD80/86^low^ Aire^−^ cells to MHCII^high^ CD80/86^high^ Aire^+^ cells. Flow cytometric analysis revealed a similar pattern of staining for both CD80 and MHCII (Fig. 4c,d). In addition, immunofluorescent staining demonstrated that, along with an overall 80% increase in the number of total mTECs (Fig. 3e, middle), the Aire^+^ population was expanded to a similar extend (Fig. 4e, left and f, left). Therefore, the activated T cells seem to induce a generally enhanced production of cells of the mTEC lineage, rather than selective expansion of specific subsets.

Our previous studies indicate that the transcriptional regulator chromobox homolog 4 (Cbx4) plays a crucial role in the development and maintenance of the thymic epithelial structure by regulating the proliferation of TEC precursors^44^. Immunofluorescence analysis demonstrated that co-culture with activated T cells led to more than 60% reduction of Cbx4^+^ cells (Fig. 4e, right and f, right). One potential explanation for this result is that over-production of mTECs causes the premature exhaustion of the limited pool of TEC progenitors.

### Excessive RANK signaling contributes to the epithelial defects induced by activated CD4^+^ T cells

**Figure 5:**
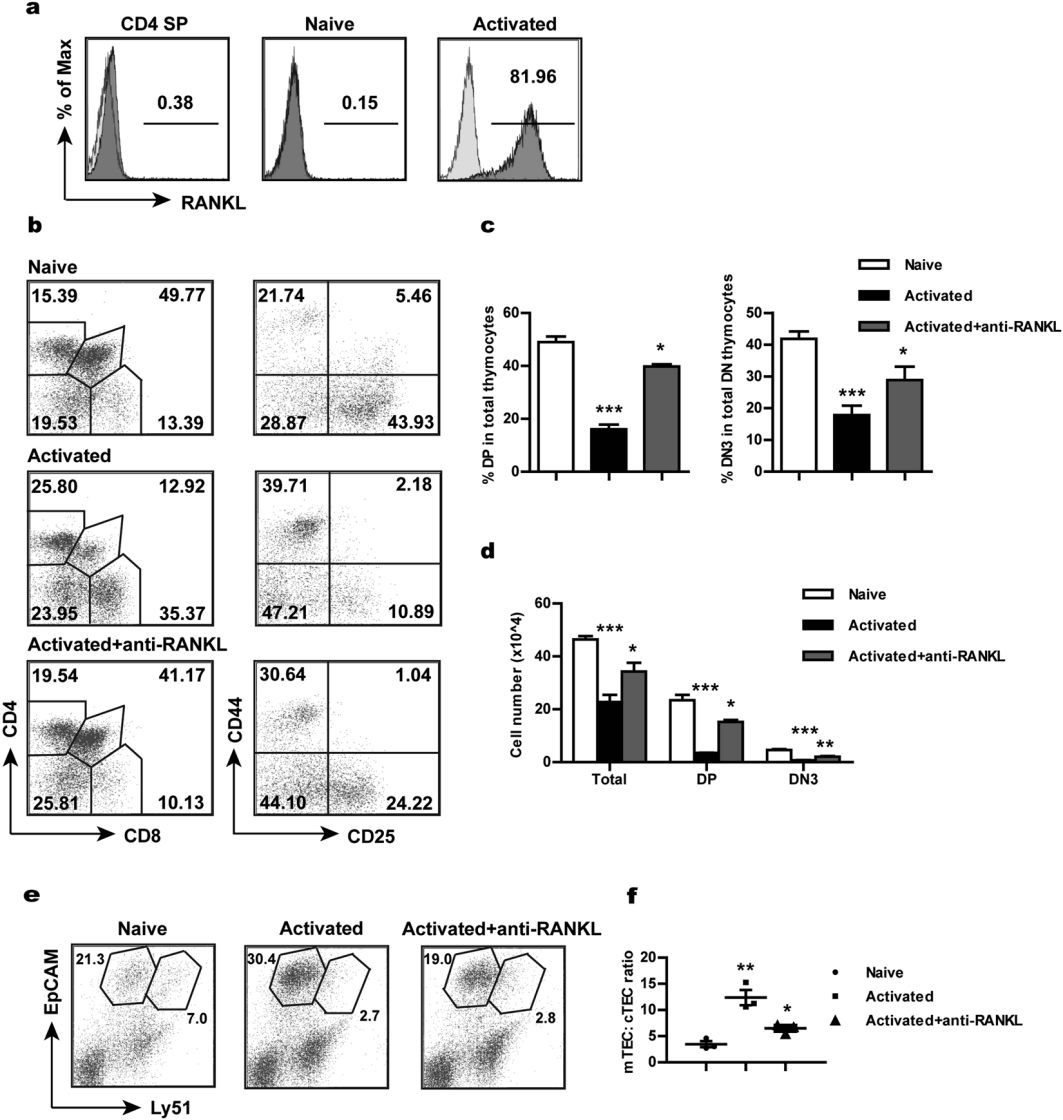
Implication of RANK signaling in activated CD4^+^ T cell-induced effects. (**a**) The expression level of RANKL measured by flow cytometry in CD4 SP thymocytes, naive CD4^+^ T cells and anti-CD3/CD28-activated CD4^+^ T cells. Shallow shades represent the staining controls. (**b**) Fetal thymuses were co-cultured with activated T cells with or without the addition of neutralising antibodies against RANKL and analysed at day 12. Dot plots show representative profiles for CD4/CD8 staining (gated on CD45.2^+^ cells) of total thymocytes and for CD25/CD44 staining (gated on CD45.2^+^ CD4^−^ CD8^−^ DN cells) of DN cells. The number indicates the percentage of cells within the gate. (**c**,**d**) The percentage of DP and DN3 cells (**c**) and the absolute number of total, DP and DN3 thymocytes (**d**) in FTOCs under different conditions. (**e**,**f**) Flow cytrometric analysis of EpCam and Ly51 expression by MACS-enriched CD45^−^ thymic stromal cells (gated on CD45^−^ cells). Representative dot plots (**e**) and the ratio of mTECs versus cTECs (**f**) are shown. The experiments were repeated three times. Each experiment included 20–30 fetal thymic lobes for each group, which were then pooled for analysis. Each dot represents the result from a single experiment. Data are presented as Mean ± SEM. *p < 0.05, **p < 0.01; ***p < 0.001.

Signaling through several members of the TNFRF, including RANK, CD40 and LTβR, is known to be critically involved in the regulation of mTEC differentiation^17, 19–23^. The signal mediated by RANK is of particular importance as its deficiency results in a marked absence of Aire^+^ cells^17, 23^. In the fetal thymus, RANK signaling is mainly triggered by RANK ligand (RANKL) expressed by lymphoid tissue inducer (LTi) cells and Vγ5^+^ dendritic epidermal T cells^23, 45^. The main sources of RANKL in the adult thymus are believed to be CD4^+^ SP thymocytes and invariant natural killer T (iNKT) cells^17, 46^. Intriguingly, high expression of RANKL was also documented in activated T cells^17, 47, 48^. We wonder whether this might contribute to the accelerated mTEC development induced by recirculating T cells. To address this possibility, we first compared RANKL expression in activated CD4^+^ and CD8^+^ T cells with that in CD4 SP thymocytes. Virtually all CD4^+^ T cells acquired RANKL expression upon activation. Moreover, the expression level in activated CD4^+^ T cells was much higher than that in CD4 SP thymocytes (Fig. 5a), suggesting that the former cells may provide stronger stimulation to developing mTECs. To a lesser extent, RANKL induction was also observed in a fraction of activated CD8^+^ T cells (Supplementary Fig. S3), which was consistent with the much less potent inhibitory effect observed for these cells (Supplementary Fig. S2).

In order to clarify the role of the highly expressed RANKL in activated CD4^+^ T cell-induced thymic changes, anti-RANKL antibodies were applied to the co-culture of fetal thymuses and activated T cells. As shown in Fig. 5b, the distorted profiles of thymocytes in the activated T cell-treated fetal thymus were largely rectified following RANKL blockade, which was accompanied by recoveries in the number of total thymocytes as well as DP and DN3 thymocytes (Fig. 5b–d). In addition, flow cytometric analysis revealed that the skewed ratio of mTECs versus cTECs was partially restored after the addition of anti-RANKL antibodies (Fig. 5e,f). Furthermore, we tested whether addition of exogenous recombinant RANKL (rRANKL) into FTOCs co-cultured with naive T cells would mimic the effect of activated T cells. Indeed, we observed similar reductions in the absolute number of total thymocytes and in the percentage of DP and DN3 cells (Supplementary Fig. S4). Taken together, these results suggest that RANKL is a key mediator for the detrimental effects induced by activated CD4^+^ T cells. Its high expression presumably results in the over-activation of the RANK signaling pathway in TECs. TEC differentiation is then heavily skewed towards the mTEC lineage as the result of excessive RANK signaling. In the long run, this impairs the thymopoiesis-supporting function of the thymic epithelium.

## Discussion

The data presented here demonstrated that thymic homing of activated CD4^+^ T cells had a profound impact on T cell development in FTOCs. In addition to an overall reduction in cellularity, activated T cells preferentially affected DP and DN3 cell production. The inhibitory effect primarily resulted from the dysfunctioning of the thymic epithelium as evidenced by the impaired capacity of the activated T cell-treated fetal thymus to support host T cell development after transplantation. Detailed analysis of the TEC compartment revealed that prolonged exposure to activated CD4^+^ T cells skewed TEC differentiation to the mTEC lineage, leading to a much enlarged mTEC population. On the other hand, diminished proliferation was observed for both mTECs and cTECs. Furthermore, we showed that excessive signaling triggered by RANKL, which was highly expressed on activated CD4^+^ T cells, played a key role in the induction of skewed differentiation to the mTEC lineage. Consistent with this notion, anti-RANKL antibodies were able to antagonise the detrimental effect of activated T cells, whereas recombinant RANKL mimicked the effect.

The present study reveals a previously unrecognised functional consequence for the re-entry of activated T cells into the thymus. For a long time, the physiological significance of thymic homing of activated T cells has remained a matter for speculation. One speculation holds that these cells could promote tolerance induction by delivering either self or foreign antigens into the thymus^38, 39^. Along the same line of thinking, recirculating T cells were proposed to support positive selection, based on findings from studies of a highly contrived model in which recirculating T cells were the only sources for ligand controlling positive selection^40^. Under normal conditions, however, positive and negative selections are known to be mediated by cTECs and mTECs in cooperation with dendritic cells^3, 4^, respectively. It is difficult to envisage how these processes could be further enhanced by non-professional, recirculating T cells. The findings from this study raise another possibility that the activated T cells homing to the thymus may serve as a mechanism to control thymic output once the peripheral T cell repertoire is established and functioning. A similar scenario has been recently reported for regulatory T (Treg) cell development^49^. Peripheral Treg cells are also capable of re-entering the thymus (which, in fact, account for a significant proportion of recirculating T cells). Once in the thymus, they exert a negative regulatory function on IL-2-dependent *de novo* differentiation of Treg cells, possibly through absorbing IL-2^49^.

The inhibition of intrathymic T cell development by recirculating T cells appears to involve a different mechanism. While a direct effect on developing thymocytes cannot be formally excluded, several lines of evidence indicate that the impaired T lymphopoiesis is most likely attributable to a dysfunctional thymic stroma. Firstly, a much severe disturbance of T cell development was observed in day 12 cultures than in day 6 cultures, arguing against an acute effect. Secondly, thymic transplantation demonstrated that the host T cell development was significantly delayed and diminished in grafts pre-cultured with activated CD4^+^ T cells compared to the control grafts, suggesting a long lasting detrimental impact on the thymic microenvironment. Thirdly, T cell development was largely restored in the activated T cell-treated fetal thymus with the addition of anti-RANKL antibodies, which presumably blocked the interaction between activated T cells and TECs. Regarding the changes in the TEC compartment, it was somehow surprising that the number of TECs was actually increased in the presence of activated T cells. More intriguingly, this increase could be solely ascribed to an expanded mTEC population while the cTEC population was found to be reduced. cTEC and mTEC are known to share a common bipotent progenitor^11, 12^. However, it is poorly understood when lineage divergence takes place and how it is regulated to maintain the balance of the two functionally diverse epithelial compartments. Worth noting, recent studies indicate that developing mTECs transverse through a transitional stage with phenotypic and molecular traits typically associated with cTECs^12, 50, 51^. In view of the intricate lineage relationship, we speculate that prolonged exposure to activated T cells results in the disruption of the delicate balance between the mTEC and cTEC lineages in differentiation, leading to overproduction of mTECs on the expenses of cTECs. cTEC defects in turn contribute to the abnormalities of T cell development.

The RANK-mediated signal plays a particularly important role in mTEC differentiation as evidenced by the much reduced or complete absence of Aire^+^ mTECs in mice deficient in RANK or RANKL^19, 23^. It is widely accepted that RANK signaling is primarily triggered by LTi cells and γδ T cells in the embryonic thymus and by SP thymocytes and iNKT cells in the postnatal thymus^17, 23, 45, 46^. The present study provides evidence that activated T cells recirculating to the thymus constitute another important source of RANKL. As a matter of fact, the very high expression of RANKL in these cells may induce excessive signaling, thereby skewing TEC differentiation to the mTEC lineage. In support of this notion, transgenic expression of soluble RANKL has been shown to increase the number of mTECs and enlarge the thymic medulla in mice^52^. Similar phenotypic changes are also documented in mice deficient in osteoprotegerin (OPG), a soluble decoy receptor for RANKL^17, 53, 54^. It will be interesting to examine whether the enhanced generation of mTECs in these animals is associated with impaired cTEC development. Nevertheless, results from this and other studies suggest that RANK signaling needs to be well adjusted to ensure proper development and function of mTECs and even cTECs.

The findings from this study have important implications for understanding the mechanisms governing thymic involution. Age-related changes in both hematopoietic and epithelial compartments have been proposed to contribute to this process. Overwhelming evidence, however, indicates that the degeneration of the thymic epithelium plays a major role. While fetal thymic grafts can be repopulated in young and aged hosts with equal efficiencies, hematopoietic progenitors from young animals fail to restore T cell development after intrathymic injection into old recipients^30^. But why does the thymic epithelium undergo degeneration early in life? Given the high turnover rate of TECs^10^, maintenance of the postnatal thymus apparently requires continuous input from a progenitor pool. Partial depletion of embryonic TEC progenitors results in reductions of thymus size in postnatal/adult stage, indicating a restricted progenitor pool^55, 56^. Furthermore, although TEC progenitors identified from fetal thymuses exhibit substantial self-renewing activity, such activity declines rapidly after birth^13^. The relatively small pool size and the limited self-renewing capacity make the epithelial compartment vulnerable to age-related deterioration as a result of progenitor exhaustion. Conceivably, the exhaustion can be accelerated if progenitors are enforced to differentiate. Indeed, premature exhaustion of epithelial stem cells was readily detected when mTEC differentiation was enforced by repetitive ablation of mature mTECs^14^. We speculate that similar mechanisms may also be functioning in activated CD4^+^ T cell-induced abnormalities of the TEC compartment. Presumably, excessive RANK signaling triggered by activated T cells drives mTEC differentiation, leading to accelerated exhaustion of the progenitor pool and ultimately to the disruption of epithelial integrity. While a detailed analysis of the TEC progenitor is lacking due to the very limited number of such cells in FTOCs, we did observe much reduced TEC proliferation and diminished numbers of Cbx4^+^ cells in activated T cell-treated cultures. Taken together, these data suggest a potential role of recirculating T cells in the early degeneration of TECs associated with thymic involution. This hypothesis is supported by the observation that thymic involution is significantly delayed in mice defective specifically in mature T cell production^57^. Further studies are warranted to investigate whether progression of thymic involution is altered in mice with similar expansion of mTECs, such as those deficient in OPG or with transgenic expression of RANKL.

## Materials and Methods

### Mice

All the animal experimental procedures were approved by the ethics committee of Peking University Health Science Center and were performed in accordance with relevant guidelines and regulations of Department of Laboratory Animal Science, Peking University Health Science Center. C57BL/6 mice were obtained from the Vital River Laboratories (Beijing, China) and raised in the animal breeding facility at Peking University Health Science Center under specific-pathogen-free conditions. Timed pregnancies were established to obtain fetal thymuses at day 16 of gestation as previously described^28^. Pregnant mice were sacrificed and the thymic lobes were isolated from the embryos.

### Antibodies and reagents

PE-Cy7-, PE-CF594-, FITC-conjugated anti-mouse CD4 (RM4-5), CD8 (53–6.7) and CD45.2 (104(RUO)); FITC-conjugated anti-mouse CD62L (MEL-14); APC-conjugated anti-mouse CD45 (30-F11); PE- and FITC-conjugated anti-mouse Ly51 (BP-1 and 6C3) were purchased from BD Biosciences. PE- and APC-conjugated anti-mouse CD25 (PC61.5) and CD44 (IM7), PE- conjugated anti-mouse MHCII (M5/114.15.2), PerCP-Cy5.5-conjugated anti-mouse CD45.1 (A20) were obtained from eBioscience. PE-Cy7-conjugated anti-mouse Epcam (G8.8) and PE-conjugated anti-mouse RANKL (IK22/5) were purchased from Biolegend; APC-conjugated anti-mouse TCRβ (H57-597) was obtained from Quantobio (Beijing, China). Rabbit anti-mouse keratin-5, rabbit anti-mouse Cbx4 and Cy5-conjugated anti-rabbit antibodies were purchased from Abcam; Alexa Fluor 594-conjugated rat anti-mouse CD326 (EpCam) (G8.8) was purchased from Biolegend; rat anti-mouse keratin-8, rabbit anti-mouse Aire, Fluorescein-UEA-1 and TRITC- conjugated anti-rat antibodies were obtained from DSHB, Santa Cruz Biotechnology, Vector Laboratories and ZSGB-BIO (Beijing) respectively. Recombinant mouse RANKL and mouse RANKL antibody were obtained from R&D systems.

### Fetal thymus organ culture (FTOC)

The hanging drop culture and FTOC were carried out as described in detail elsewhere^58^. Briefly, thymic lobes were obtained from day 16 fetuses. FACS sorted CD4^+^ naive T cells from C57BL/6 mice were activated *in vitro* by stimulating with 2 μg/ml anti-CD3 (145-2C11) and 1 μg/ml anti-CD28 (37.51) for 48 h. Hanging drop cultures were prepared in Terasaki plates by mixing the fetal thymus with 2 × 10^4^ activated or naive CD4^+^ T cells in 20 μl RPMI 1640 medium supplemented with 15% fetal bovine serum (Biochrom Ag, Berlin). The plates were then inverted to form hanging drops and incubated in 5% CO_2_ at 37 °C for 24 h. After incubation, the lobes were washed in fresh RPMI 1640 to remove free activated or naive T cells away. Then the fetal thymuses were put on 0.45 μm Mixed Cellulose Ester Gridded Filters (HAWG 01300, Merck Millipore) on the top of cut Absorbable Gelatin Sponges (Jinling Pharmaceutical) in 12-well plates filled with culture medium. The lobes were cultured for 6 or 12 days in the incubator (5% CO_2_, 37 °C) and were then subjected to further analysis. Neutralization antibody and recombinant protein of RANKL were used in 5 μg/ml and 1 μg/ml respectively. The agents were given from the start of the hanging drop culture and throughout the full course of FTOC. Cultures were fed every 2–3 days and the agents were given together with the fresh culture medium.

### Flow cytometry analysis and cell sorting

Thymic lobes were carefully removed from filters at the indicated time and washed gently in PBS. To make single-cell suspensions, the lobes were digested with collagenase/dispase (Roche) and DNase I (Roche) by incubating at 37 °C for 15 min with vortexing every 5 min. Cells were centrifuged in cold PBS with the addition of 0.5 mM EDTA to prevent the aggregate formation. For T cell analysis, cells were directly stained with fluorochrome-labeled antibodies on ice for 30 min. For TEC analysis, cells were first enriched by depleting CD45^+^ thymic cells using a MACS immunomagnetic cell sorter (Miltenyi) according to manufacturer’s protocol. The enriched TECs were stained with fluorochrome-conjugated mouse antibodies on ice for 30 min. Flow cytometry was then conducted on a Beckman FACS Galios (Beckman Coulter) and data analysis was performed using Kaluza software.

To collect the naive CD4^+^ (or CD8^+^) T cells, lymphoid cells prepared from lymph nodes of C57BL/6 mice were stained with fluorochrome-conjugated antibodies and CD4^+^ CD8^−^ CD44^low^ CD62L^high^ (or CD4^−^ CD8^+^ CD44^low^ CD62L^high^) cells were sorted using a BD FACS Aria II.

### Kidney capsule transplantation

The kidney capsule transplantation was performed as previously described^30^. After intra-peritoneal administration of pentobarbital sodium (5 mg/ml), a small centrodorsal incision was made to expose the kidney. Thymic lobes harvested from activated or naive T cell-treated FTOCs at day 12 were placed under the left and right kidney capsule, respectively. The incisions were then closed with sterile sutures. Development of host-derived thymocytes in the grafted thymuses was analyzed by flow cytometry at indicated time points after surgery.

### Immunofluorescence microscopy

Fetal thymuses were carefully removed from filters at day 12 of FTOC and washed in PBS. As the cultured thymic lobe was too thin to be sliced, immuno-staining was performed with the whole lobe. They were fixed in 4% paraformaldehyde (PFA) (Dingguochangsheng Biotechnology, Beijing) for 20 min and blocked with 5% bovine serum albumin at 37 °C for 1 h. Lobes were then incubated at 4 °C overnight with primary antibodies and Hoechst33342. After 1 h incubation with secondary antibodies, the thymic lobes were placed on glass slides and mounted in Vectashield mounting medium (Vector Laboratories). Images were taken with Leica TCS SP5 or SP8 microscopes. For quantification analysis, 10 randomly chosen sections were counted using Image J.

### Brdu incorporation by TECs

Brdu was added to the culture medium of FTOC on day 11 at a final concentration of 10 μM. After incubation for additional 24 h, the thymic lobes were harvested and processed to obtain single cell suspension. Following depletion of CD45^+^ cells by MACS, cells were stained for a combination of surface markers. Brdu incorporation was subsequently detected using the Brdu flow kit (BD Biosciences) following the manufacturer’s protocol.

### TUNEL assay

Thymic lobes were harvested from day 12 cultures. TEC preparation and surface staining were performed as described above. The apoptotic cells were detected using *In Situ* Cell Death Detection kit (Roche Diagnostics) according to the manufacturer’s instructions.

### Statistical analysis

Data are presented as mean ± SEM. Statistical significances were assessed by Student’s t test or two-way Anova (for multiple variant comparisons) using GraphPad Prism software (GraphPad).

## Electronic supplementary material

Supplementary Figures and Legends
