# Supplementary material for: Thymic homing of activated CD4+ T cells induces degeneration of the thymic epithelium through excessive RANK signaling
Source: Sci Rep. 2017 May 25;7:2421. doi: 10.1038/s41598-017-02653-9 (PMC5445095; doi:10.1038/s41598-017-02653-9)
Supplement: Supplementary file 1 — Supplementary Figures and Legends [file 41598_2017_2653_MOESM1_ESM.doc]

**Supplementary Informations**

**Thymic homing of activated CD4^+^ T cells induces degeneration of the thymic epithelium through excessive RANK signaling**

Chen Yin^a#^, Xiao-Yan Pei^a#^, Hui Shen^a^, Ya-Nan Gao^a^, Xiu-Yuan Sun^a^, Wei Wang^a^, Qing Ge^a^ and Yu Zhang^ab*^

^a^Department of Immunology, School of Basic Medical Sciences, Key Laboratory of Medical Immunology of Ministry of Public Health, Peking University Health Science Center, Beijing, China

^b^Institute of Biological Sciences, Jinzhou Medical University, Jinzhou, Liaoning, China

#These authors contribute equally to this work.

*To whom correspondence should be addressed at:

Yu Zhang, Department of Immunology, Peking University Health Science Center, 38 Xue Yuan Road, Beijing, 100191, China, Tel: 0086-10-82802593, Fax: 0086-10-82801436, E-mail: zhangyu007@bjmu.edu.cn

**Supplementary Figure Legends**

**Supplementary Fig. S1: Entry of activated CD4^+^ T cells into the fetal thymus and their impact on T cell development in day 6 cultures.** Fetal thymuses (CD45.2^+^) were co-cultured with 2x10^4^ naive or activated CD4^+^ T cells (CD45.1^+^) in hanging drops for 24 hours. FTOCs were then set up and harvested at day 6 or day 12. (a) The percentage of CD45.1^+^ T cells identified by flow cytometry in day 12 cultures. (b) Flow cytometric analysis of T cell development in FTOC at day 6. Dot plots show representative profiles for CD4/CD8 staining (gated on CD45.2^+^ cells) and for CD25/CD44 staining (gated on CD45.2^+^ CD4^-^ CD8^-^ DN cells). (c, d) The percentage of DP (left) and DN3 (right) cells (c) and the absolute number (d) of total, DP and DN3 thymocytes. The experiments were repeated four times with 3- 5 thymic lobes for each group in each experiment. Data are presented as Mean ± SEM. *, p < 0.05; **, p < 0.01; and ns, not significant*.*

**Supplementary Fig. S2: Modest effect of activated CD8^+^ T cells on T cell development in FTOCs.** FTOCs were set up with d16 fetal thymuses pre-incubated with 2x10^4^ naive or activated CD8^+^ T cells (CD45.1^+^). The cultures were harvested and analysed at day 12. (a) Thymic entry of CD45.1^+^ T cells as measured by flow cytometry at day 12. (b) The total number of thymocytes recovered from a single thymic lobe. (c) Flow cytometric analysis of T cell development in CD8^+^ T cell treated-FTOCs. Dot plots show representative profiles for CD4/CD8 staining (gated on CD45.2^+^ cells) of total thymocytes and for CD25/CD44 staining (gated on CD45.2^+^ CD4^-^ CD8^-^ DN cells) of DN cells. The number indicates the percentage of cells within the gate. (d) The percentage of DP (upper) and DN3 (lower) thymocytes. The experiments were repeated three times with 3- 5 thymic lobes for each group in each experiment. Data are presented as Mean ± SEM. *, p < 0.05; ns, not significant.

**Supplementary Fig. S3: Comparison of RANKL expressions in CD4^+^ and CD8^+^ activated T cells.** Naive or anti-CD3/CD28 activated CD4^+^ and CD8^+^ T cells were stained with anti-RANKL antibody and analysed by flow cytometry. (a) Representative histograms. Shallow shades represent the staining controls. (b) The percentage of RANKL^+^ cells. The experiments were repeated three times and data are presented as Mean ± SEM. **, p < 0.01; ***, p < 0.001.

**Supplementary Fig. S4:** **Recombinant RANKL mimics the effect of activated CD4^+^ T cells.** Exogenous recombinant RANKL was added to FTOCs co-cultured with naive CD4^+^ T cells. T cell development was analysed at day 12. (a) Representative profiles for CD4/CD8 staining (gated on CD45.2^+^ cells) of total thymocytes and CD25/CD44 staining (gated on CD45.2^+^ CD4^-^ CD8^-^ DN cells) of DN cells. (b) The percentage of DP (left) and DN3 (right) cells. (c) The absolute number of total, DP and DN3 thymocytes. The experiments were repeated three times with 3- 5 thymic lobes for each group in each experiment. Data are presented as Mean ± SEM. ***, p < 0.001; ns, not significant*.*

**Fig.S1**

**
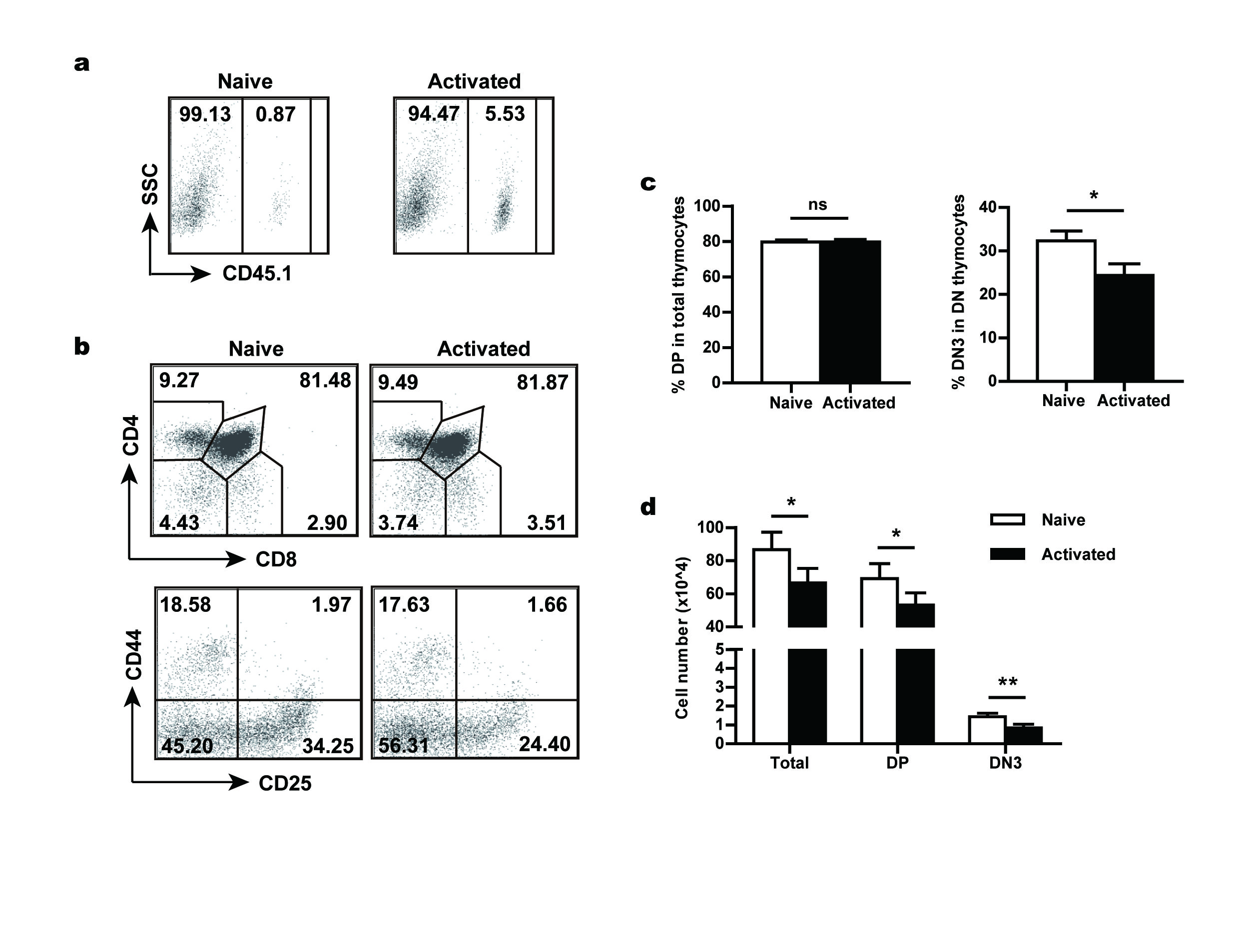
**

**Fig. S2**

**
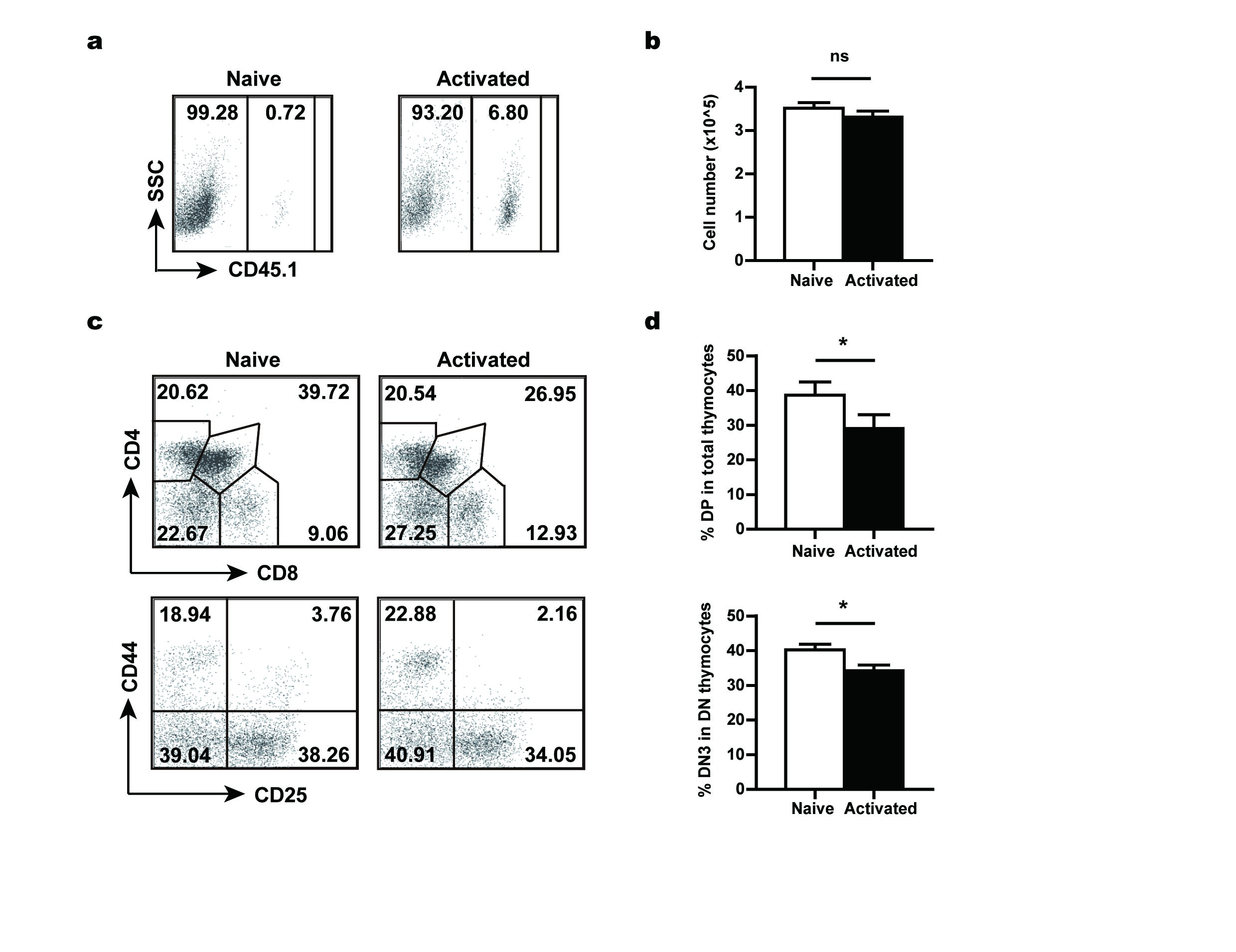
**

**Fig. S3**

**
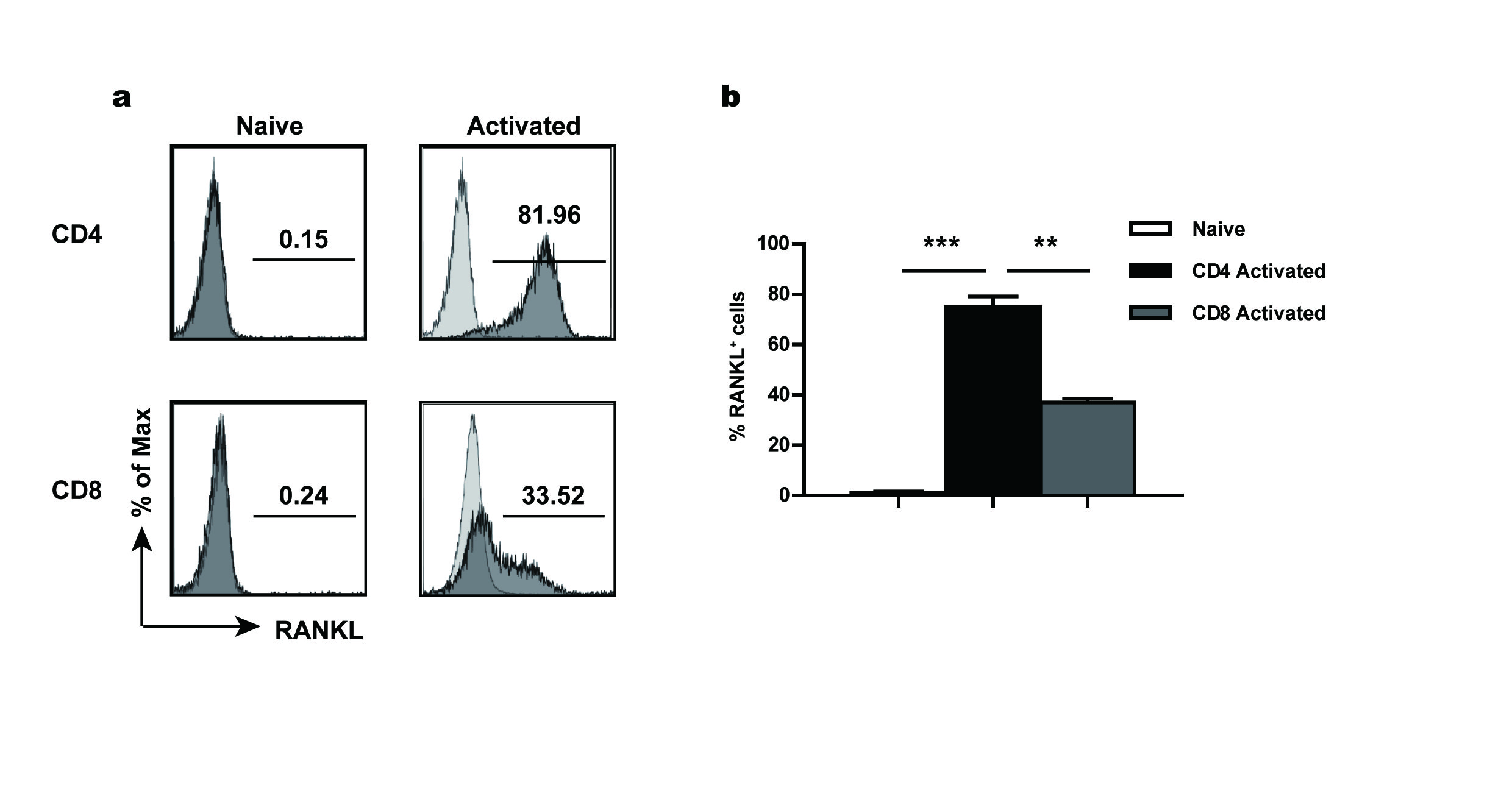
**

**Fig. S4**

**
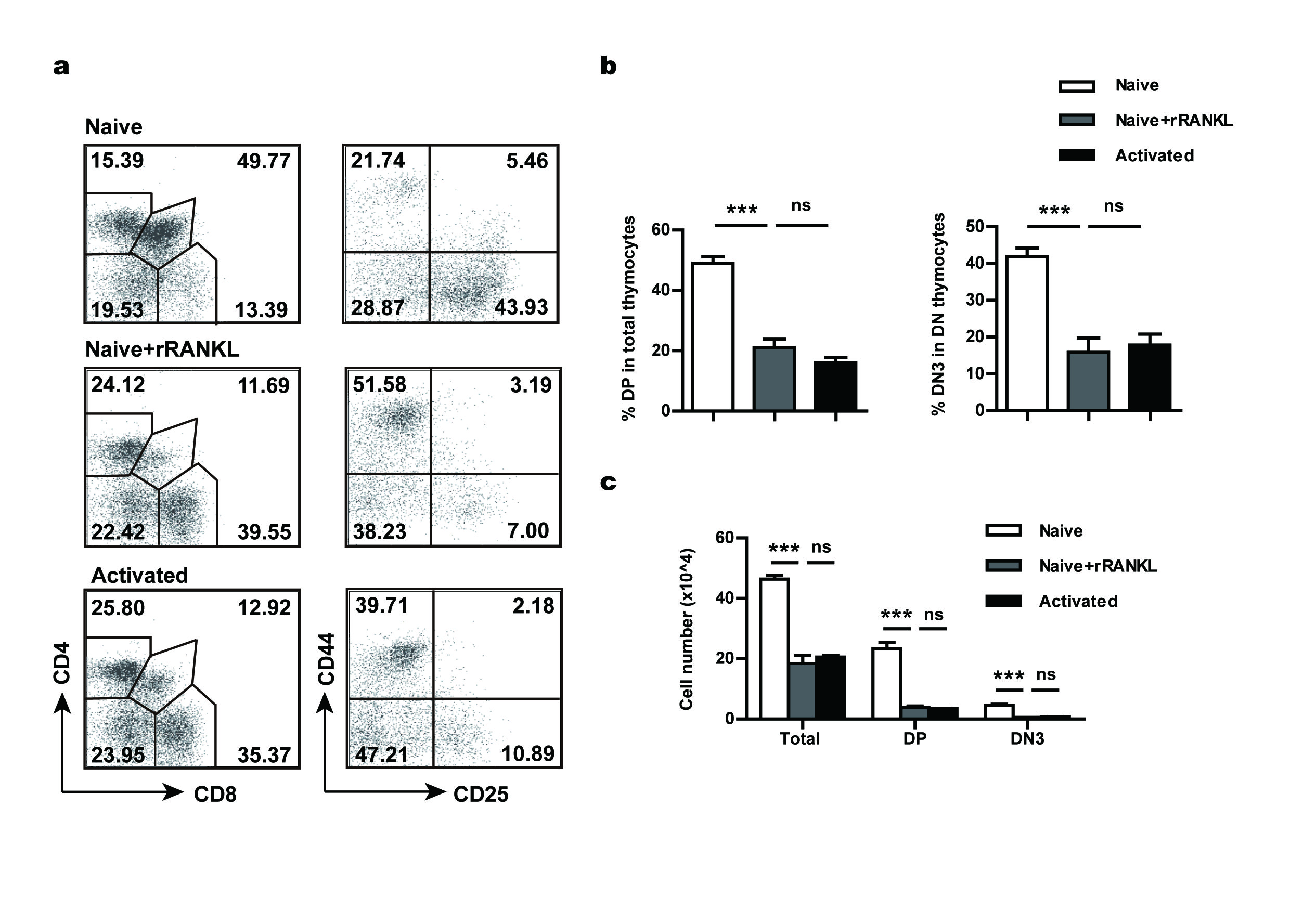
**
